# The double histone fold: Structure, functional implications across the tree of life and relevance to protein design

**DOI:** 10.1002/pro.70573

**Published:** 2026-04-17

**Authors:** Anna Ranaudo, Toshiko Miyake, Ugo Cosentino, Claudio Greco

**Affiliations:** ^1^ Department of Earth and Environmental Sciences University of Milano‐Bicocca Milan Italy; ^2^ Department of Pharmaceutical and Pharmacological Sciences University of Padova Padova Italy

**Keywords:** DNA‐binding proteins, double histone fold, histone pseudodimer, protein design, protein evolution, structural convergence

## Abstract

The histone fold is one of the most ancient and versatile structural motifs in protein biology, best known for its role in chromatin organization. A remarkable variation of this motif is the double histone fold (DHF), in which two histone folds are encoded within a single polypeptide chain and assemble intramolecularly into a histone‐like pseudodimer. Initially considered a rare structural anomaly, the DHF has now been identified in archaea, viruses, bacteria and eukaryotes, often in proteins unrelated to canonical chromatin architecture. In this review, we analyze the DHF from a protein engineering and evolutionary perspective, focusing on its structural determinants, modularity and repeated emergence across distant lineages. We discuss how the DHF exemplifies a robust protein design solution for DNA binding and macromolecular interaction, shaped by both divergent and convergent evolution. Finally, we highlight implications for protein design, including the DHF as a natural template for engineering DNA‐binding modules that can be used, for example, to modulate chromatin organization.

## 1 INTRODUCTION

Histones are fundamental components of chromatin, forming the protein core around which DNA is wrapped in eukaryotic nuclei. The canonical nucleosome is built from core histones—two copies each of H2A, H2B, H3, and H4—organized as heterodimers that form an octameric particle, around which ~147 bp of DNA is wrapped (Luger et al., 1997). At the structural level, this organization relies on the histone fold, a conserved motif composed of three α‐helices connected by two loops, which mediates dimerization through extensive hydrophobic interactions (Baxevanis & Landsman, 1997; Ruiz‐Carrillo et al., 1979).

**FIGURE 1:**
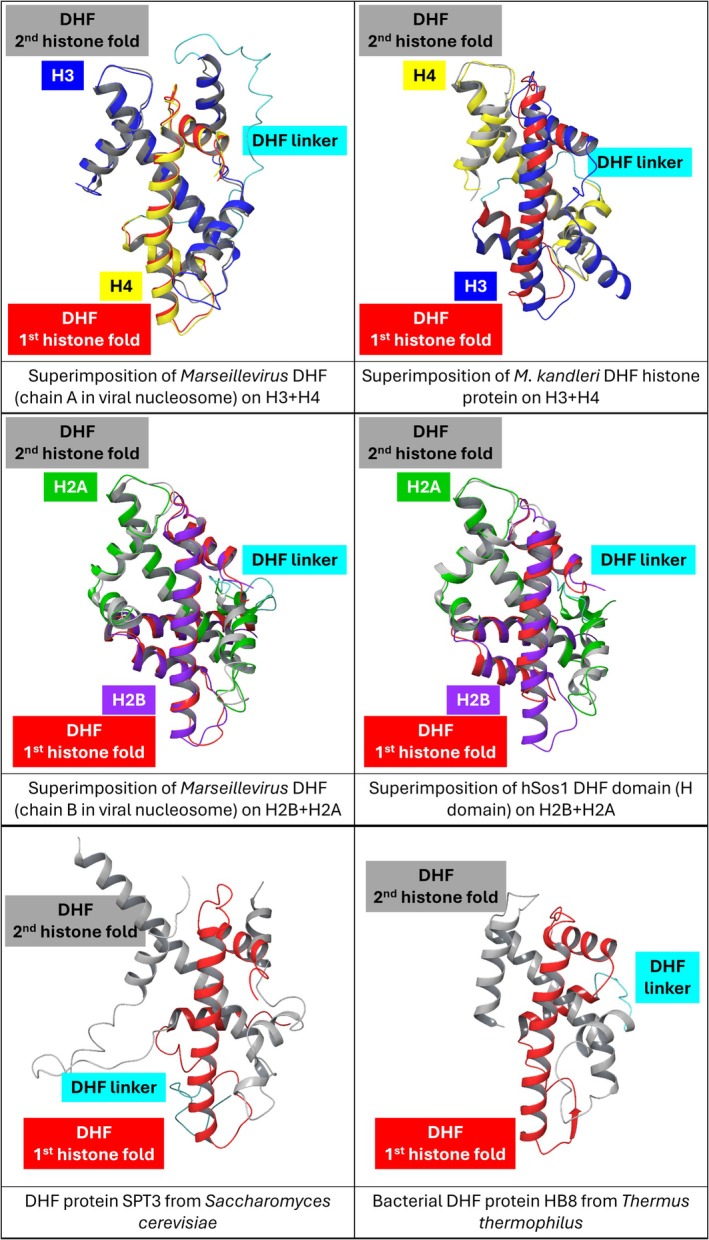
Overview of experimentally determined structures available for the various DHF types listed in Tables 1 and 2. The tags “1st histone fold” and “2nd histone fold” refer to the position of the two histone folds along the DHF protein sequence, for each of the six DHF proteins represented; the linker joining together histone folds is also highlighted. In all cases in which a DHF protein shares significant sequence identity with the components of a nucleosomal histone dimer (H2A–H2B or H3–H4), the structure of the DHF was superimposed with the crystal structure of the relevant dimer (PDB ID: 1AOI). The DHF structures pictured are the following, from top‐right to bottom‐left: *Marseillevirus* H3–H4‐like DHF (PDB ID: 7N8N, chain A); *Methanopyrus kandleri* DHF histone protein (PDB ID: 1F1E); *Marseillevirus* H2A–H2B‐like DHF (PDB ID: 7N8N, chain B); H domain of hSos1 (PDB ID: 1Q9C); SPT3 protein from *Saccharomyces cerevisiae* (PDB ID: 6T9I); HB8 bacterial DHF protein from *Thermus thermophilus* (PDB ID: 1WWI).

**FIGURE 2:**
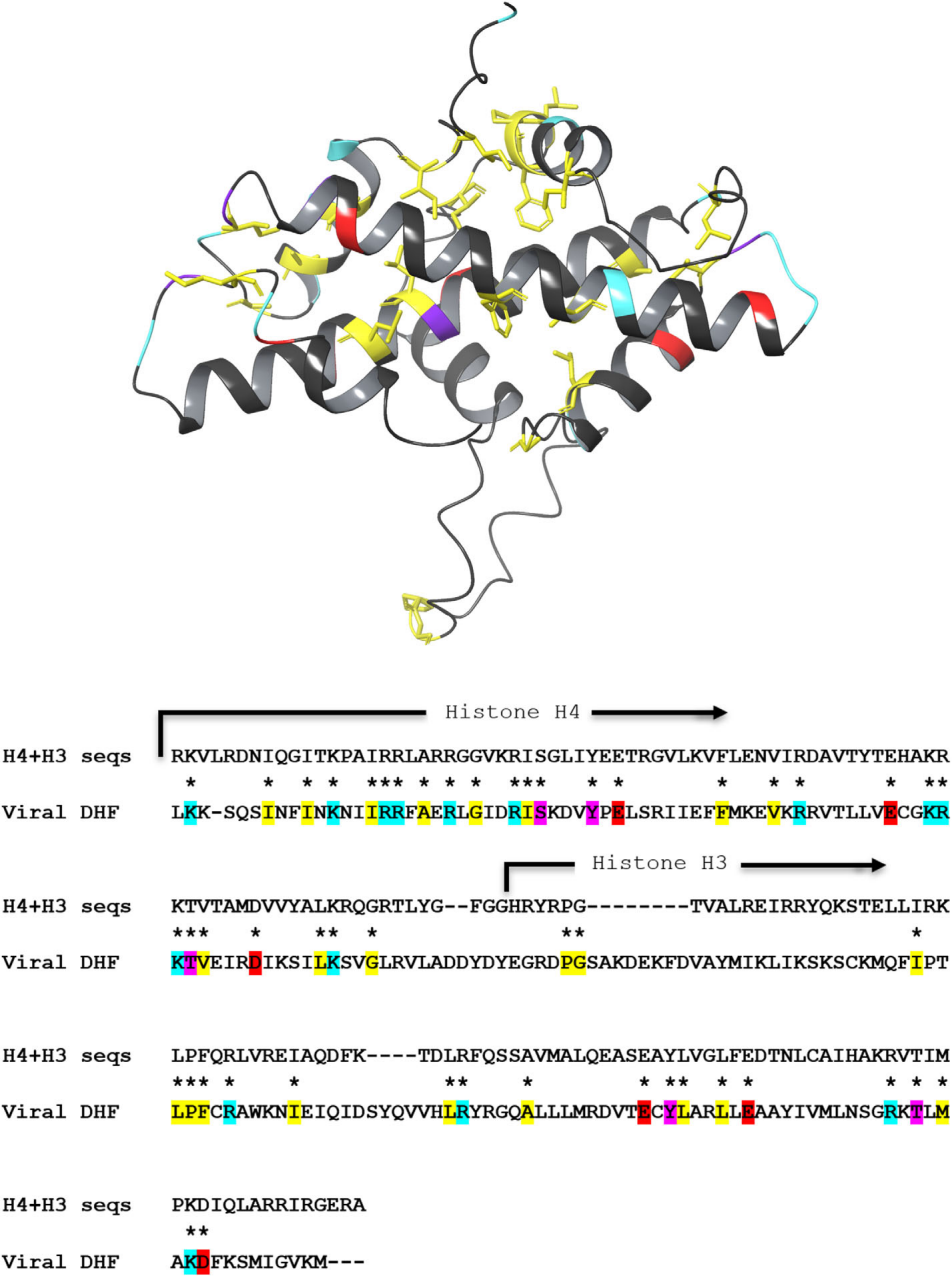
Alignment between the primary amino acid structure of human histones H4 and H3 and the amino acid sequence of the double histone fold from *Heliothis zea* nudivirus 1 (previously known as Hz‐1 virus) (Greco, Fantucci, & de Gioia, 2005), and cartoon representation of the AlphaFold 3D model of the viral pseudodimer. In the sequence alignment, the highlighted residues are conserved and are colored as follows: Yellow, non‐polar residues; pink, polar residues; cyan, basic residues; red, acidic residues. The same colors were used to color the cartoon representation of the 3D model backbone, in correspondence to the conserved residues; only for the non‐polar conserved residues, the sidechains are shown and colored in yellow as well.

Although historically associated with eukaryotic chromatin, histone folds are far more widespread than initially thought. Archaeal histones (Sandman et al., 1990; Schwab et al., 2025), bacterial histone‐like proteins (Alva & Lupas, 2019; Hu et al., 2025; Schwab et al., 2025), viral histones (Schwab et al., 2025; Yoshikawa et al., 2019), and numerous histone fold–containing domains in non‐chromatin proteins (Birck et al., 1998; Xie et al., 1996) illustrate how this structural motif has been repeatedly reused during evolution. Among these variations, the double histone fold (DHF) represents a particularly striking example. As shown in Figure 1, which reports the crystallographically or microscopically known variants of the DHF assembly, this configuration features two histone folds consecutively encoded within a single polypeptide that fold together intramolecularly to form a structure closely resembling a histone heterodimer. Conservation of the hydrophobic core typical of canonical histone dimers is usually observed in DHF proteins; an example in such regard is shown in Figure 2, which reports the amino acid sequence as well as the modeled 3D structure of a viral DHF protein featuring regions of sequence similarity to nucleosomal histones H3 and H4 (Greco, Fantucci, & de Gioia, 2005).

The first experimentally characterized example of the DHF came from an archaeal histone expressed by *Methanopyrus kandleri* (Fahrner et al., 2001; Slesarev et al., 1998). Such striking discovery immediately inspired investigations in quite different areas of research, that is, in the study of eukaryotic transcription factor subunits such as the SPT3 protein—a component of the SAGA complex (Grant et al., 1997)—that was found to contain two histone folds on the same amino acid sequence (Birck et al., 1998). Researchers hypothesized that a histone pseudodimer could form also in the case of SPT3, although an alternative picture based on intermolecular interactions between histone folds belonging to two different SPT3 proteins was not excluded (Birck et al., 1998); the latter possibility would make the tandem repeat become a platform for the dimerization of the transcription factor. The first hypothesis turned out to fit well crystallographic evidences that were published only recently (Herbst et al., 2021; Wang et al., 2020), showing that such component of the SAGA complex is a DHF protein in which one of the two histone folds has an unusually long central helix (see the *Saccharomyces cerevisiae* SPT3 protein structure in Figure 1); notably, the alternative hypothesis has not found any experimental support to date; more generally speaking, up to now no protein featuring two tandem histone folds was found to be able to exploit such motif for homodimerization. In other words, data that accumulated over the last quarter of a century in literature always associate newly discovered couples of histone folds in tandem repeat with intramolecular pseudodimers formation.

Over the past two decades, computational and structural studies have uncovered additional DHF‐containing proteins in eukaryotes, viruses, and bacteria, progressively reshaping our understanding of the prevalence and functional versatility of this fold (Dulmage et al., 2015; Greco, Sacco, et al., 2005; Irwin & Richards, 2024; Liu et al., 2021; Schwab et al., 2025; Sondermann et al., 2003; Valencia‐Sánchez et al., 2021). Recent large‐scale analyses further indicate that the DHF may be much more widespread than previously appreciated, with evidence of extensive convergent evolution toward this architecture (Alva & Lupas, 2019; Miyake et al., 2026).

In this review, we synthesize current knowledge on the double histone fold, focusing on three main aspects: (i) its structural definition and relationship to canonical histone dimers, (ii) its phylogenetic and taxonomic distribution, and (iii) its functional roles, particularly in DNA binding and genome organization. By integrating structural biology, bioinformatics, and evolutionary perspectives, we aim to provide a coherent framework for understanding the biological significance of the double histone fold.

## 2 STRUCTURAL BASIS OF THE DOUBLE HISTONE FOLD

### 2.1 The canonical histone fold

The histone fold is a compact *α*‐helical motif consisting of a long central helix flanked by two shorter helices (see Figure 1). In canonical histones, the fold mediates heterodimerization through a handshake‐like arrangement in which the long central helices pack antiparallel to each other, while the shorter flanking helices contribute to stabilization and DNA contacts (Luger et al., 1997). This architecture underlies the formation of H2A–H2B and H3–H4 heterodimers, which are the building blocks of the nucleosome.

A key feature of the histone fold is the conservation of hydrophobic residues at specific positions along the helices, ensuring proper packing and stability of the dimer. Positively charged residues, particularly on the N‐terminal tails and on exposed surfaces of the fold, mediate interactions with DNA (Figure 2).

### 2.2 Definition of the double histone fold

In the double histone fold, two histone fold motifs are present sequentially in the same polypeptide chain and fold together intramolecularly to form a pseudodimer that closely mimics the structure of a canonical histone heterodimer (Greco, Sacco, et al., 2005; Slesarev et al., 1998; Sondermann et al., 2003). Structural studies have shown that the relative orientation of the two folds, as well as the pattern of hydrophobic interactions, is in general remarkably similar to those observed in nucleosomal dimers (Fahrner et al., 2001; Greco, Sacco, et al., 2005; Sondermann et al., 2003).

The linker connecting the two histone folds plays a critical role in enabling proper folding. Its length and flexibility must accommodate the spatial arrangement required for dimer‐like packing. Interestingly, different DHF proteins display different orders of histone‐like domains (e.g., H2B‐like followed by H2A‐like, or vice versa), implying distinct linker constraints and possibly different evolutionary origins (Irwin & Richards, 2024; Miyake et al., 2026).

### 2.3 Structural mimicry and divergence

**FIGURE 3:**
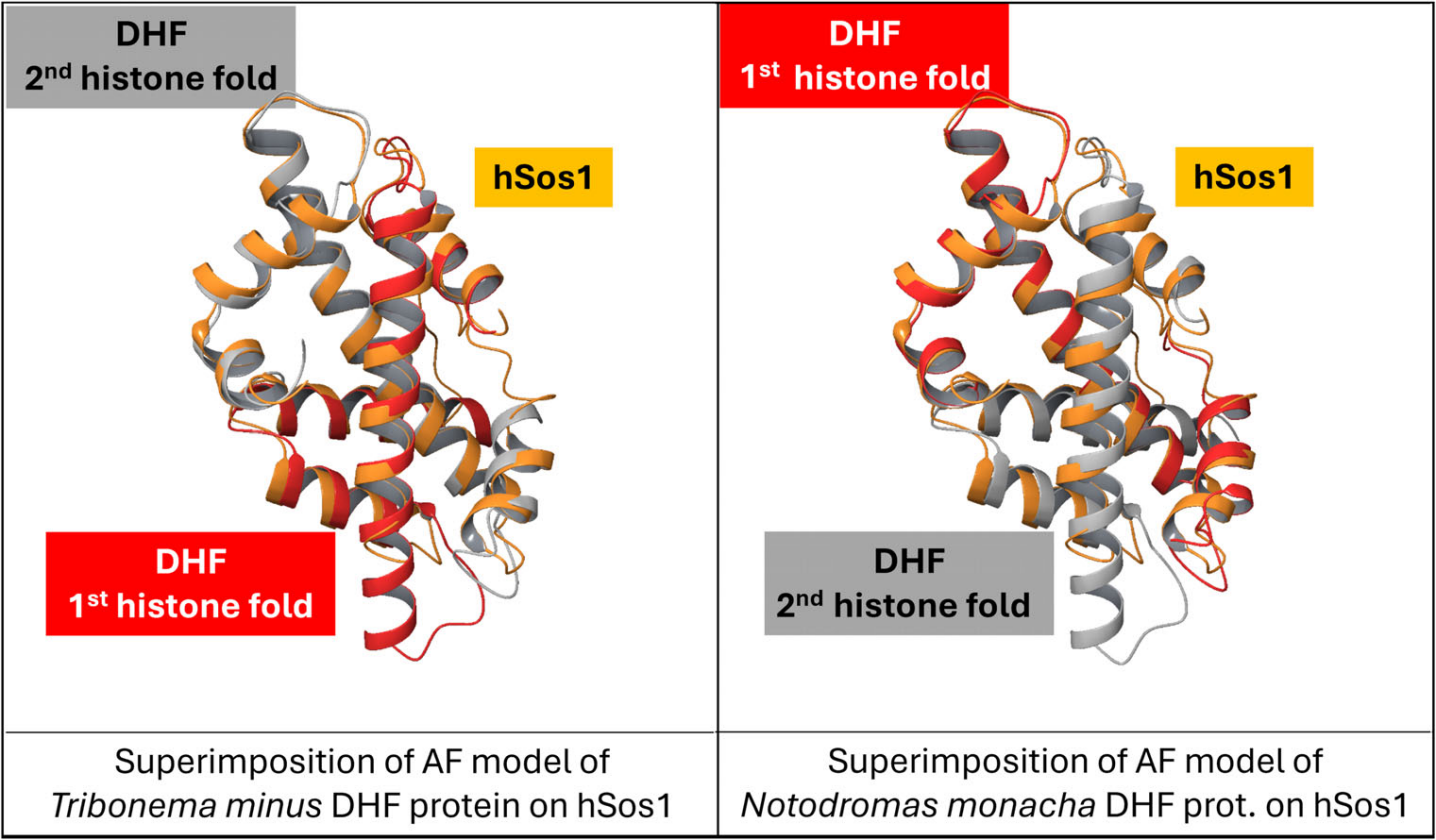
Superimposition of the crystal structure of hSos1 H domain with AlphaFold models of the HSIH‐DHF proteins from *T. minus* (left half of the figure) and from *N. monacha* (right half).

One of the most striking aspects of the DHF is the degree of structural conservation despite substantial sequence divergence (Fahrner et al., 2001). For example, the N‐terminus of human Sos1 (hSos1) includes an H2B‐like fold followed by an H2A‐like fold that constitute the so called “H domain”; this is a histone pseudodimer showing very low sequence identity to canonical human histones (Sondermann et al., 2003; Wang et al., 2020)—ca. 22% and 23% with histones H2B and H2A, respectively—yet its three‐dimensional structure is almost superimposable on an H2A–H2B canonical dimer (Figure 1) (Greco, Sacco, et al., 2005; Sondermann et al., 2003). Such DHF domain in hSos1 shares very similar 3D structure also with a group of recently identified “High Sequence Identity with Histones” (HSIH, see Table 1 and vide infra) DHF proteins composed of an H2B‐like domain followed by an H2A‐like domain (see Figure 3, left‐hand side) (Miyake et al., 2026). This highlights the robustness of the DHF architecture and its tolerance to sequence variation, provided that key hydrophobic and geometric constraints are maintained. Actually, at the histone fold level, low sequence similarity together with strong structural resemblance is strong evidence in support of the homology relationship; nevertheless, this does not imply a single evolutionary origin of the overall DHF architecture, which instead appears to have emerged independently multiple times across distinct lineages. For example, the DHF domain in hSos1 shares a high degree of structural similarity also with HSIH‐DHF proteins whose H2A‐like domain is followed by an H2B‐like domain, which is a swapped disposition of the two histone folds along the amino acid sequence as compared to the case of hSos1 (superimposition of structures shown in Figure 3, right‐hand side). Clearly, the DHF domain in hSos1 and in the HSIH‐DHF protein just mentioned must have originated as a result of independent gene fusion events.

**TABLE 1:** Proteins featuring the double histone fold in cellular life.

DHF variant or family	Expressed in	References	PDB IDs of experimentally‐determined structures (*species*)
*M. kandleri*‐type and *Halobacteria*‐type histone doublets	Archaea (kingdoms: Asgard archaea; Methanobacteriati)	Fahrner et al. (2001), Stevens and Warnecke (2023)	1F1E (*M. kandleri*)
H domain in Sos proteins	Eukaryotes (kingdom Animalia); found also in the protist *Capsaspora owczarzaki*	Greco, Sacco, et al. (2005), Sondermann et al. (2003), Iversen et al. (2014), Huang et al. (2019), Sacco et al. (2012)	1Q9C, 3KSY (*H. sapiens*)
ABTB2‐type DHF proteins	Eukaryotes (kingdom Animalia)	Greco, Sacco, et al. (2005), Hayashi et al. (1997), Iqbal et al. (2022), Gong et al. (2020)	n/a
SPT3 proteins	Eukaryotes^a^ (kingdoms: Animalia, Fungi; Protists)	Wang et al. (2020), Herbst et al. (2021)	6T9I (*S. cerevisiae*); 7KTR (*H. sapiens*)
Bacterial DHF proteins	Bacteria^b^(kingdoms: Thermotogati, Pseudomonadati, Bacillati)	Qiu et al. (2006)	1WWI (*T. thermophilus*); 1R4V (*A. aeolicus*)
HSIH‐DHF proteins	Eukaryotes (kingdom: Animalia)	Miyake et al. (2026)	n/a

^a^ Homologs of SPT3 are present also in plants, however they are in general much shorter than their counterparts in non‐photosynthetic eukaryots, and are unlikely to be DHF proteins.

^b^ Bacteria‐type DHF proteins are also found in some archaea (Qiu et al., 2006; Stevens & Warnecke, 2023), probably due to horizontal gene transfer (see Section 3.3).

## 3 EVOLUTIONARY DISTRIBUTION OF THE DOUBLE HISTONE FOLD

### 3.1 Early discovery of the double histone fold: The histone of the archaeon *M. kandleri*

**FIGURE 4:**
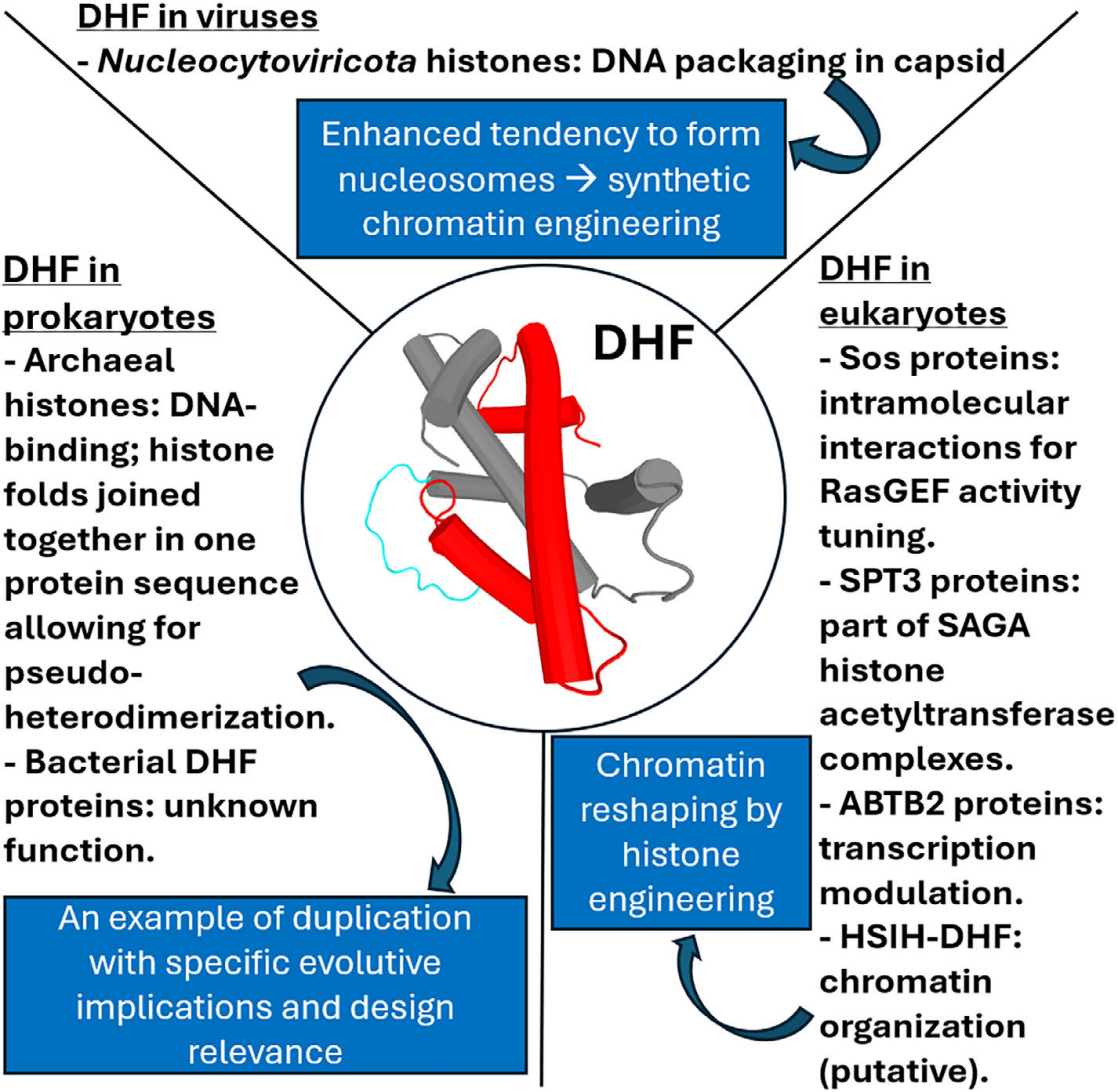
Schematic outlook on DHF proteins with respect to distribution, function, and design‐related issues.

As mentioned above, the first structural evidence for a DHF came from archaeal histones in 2001 (Fahrner et al., 2001). The histone from the hyperthermophile microorganism *M. kandleri* (HMk, see Figure 1) was shown to contain two histone folds within a single polypeptide, forming a stable pseudodimer capable of binding DNA (Musgrave et al., 2000). Given the relative simplicity of archaeal chromatin organization, this finding suggests that intramolecular histone dimerization might represent an ancient solution for DNA compaction, also in relation with the special growth requirements of *M. kandleri* that is among the most heat‐tolerant of hyperthermophilic prokaryotes (it can live and reproduce at temperatures as high as 122°C) (Takai et al., 2008). Indeed “fusion” of two proteins can increase the barrier for unfolding, making the protein more stable than the corresponding dimer (Finch & Kim, 2018); in fact, genes fusion to form multi‐domain proteins can favor overall stability by reducing unfolding surfaces and promoting cooperative folding (see also Section 5 and Figure 4).

As far as the analysis of amino acid sequence of HMk is concerned, the two histone folds that compose such DHF protein are 28% identical to each other; moreover, they are ∼21% identical in amino acid sequence to other histone proteins, including nucleosome core histone proteins (Fahrner et al., 2001). Notably, high structural similarity can be evidenced when the x‐ray structures of HMk and of a eukaryotic histone pair, such as the H3‐H4 heterodimer, are superimposed (Figure 1).

DHF proteins of HMk type were found in two different classes within the Methanobacteriati kingdom, that is, in Methanopyri—a class that includes *M. kandleri*—and also in species within the Methanobacteria class (see Table 1). Interestingly, *M. kandleri*‐type DHF was also found in Asgard archaea, which is particularly intriguing given the fact that the Asgard kingdom includes the archaea species more closely related to eukaryotes (Zaremba‐Niedzwiedzka et al., 2017); implications of such occurrence of archaeal DHF from a functional perspective are more thoroughly discussed below, in Section 4.

Finally, recent literature evidenced the presence of histone doublets in Halobacteria species, which also belong to Methanobacteriati and are distantly related to *M. kandleri* (Stevens & Warnecke, 2023). These proteins were previously referred to as the Halobacteria‐type doublets (Stevens & Warnecke, 2023); such classification is adopted in the present review as well (see Table 1). The very sparse recurrence of DHF among distantly related archaeal lineages—as shown in phylogenetic analyses by Stevens and Warnecke (2023)—is indicative of convergent evolution as an active process leading to the emergence of DHF variants also within prokaryotes.

### 3.2 Viruses encoding DHF proteins

One of the most compelling demonstrations of the functional relevance of the DHF comes from viruses. Several large DNA viruses, particularly members of the nucleocytoplasmic large DNA viruses (NCLDV, corresponding to the Nucleocytoviricota phylum), encode histone‐like proteins, including double histone folds (Liu et al., 2021; Thomas et al., 2011; Toner et al., 2024).

In fact, in addition to the abovementioned DHF found in Heliothis zea nudivirus 1 (see Figure 2 and Table 2), DHF proteins are common among NCLDVs belonging to the early‐branching superclades within the Nucleocytoviricota phylum (Irwin & Richards, 2024), such as marseilleviruses, iridoviruses and medusaviruses. The histone folds constituting such DHF proteins were found to have significant amino acid sequence similarity to both eukaryotic and archaeal histones; in particular, sequence identity to eukaryotic histones was found to be <30% (Liu et al., 2021). Very interestingly, cryogenic electron microscopy investigations showed that H2A–H2B‐like and H3–H4‐like doublets in marseilleviruses assemble with DNA to give place to nucleosome particles (Liu et al., 2021; Valencia‐Sánchez et al., 2021). Such particles can form not only in vitro but also inside the viral capsid, thereby promoting genome compaction within virions (Bryson et al., 2022). The intramolecular nature of the DHF may be particularly advantageous in the viral context, where genome size and protein economy are under strong selective pressure. The presence of DHFs in viruses raises important evolutionary questions, including whether these proteins were acquired from hosts via horizontal gene transfer or evolved independently as convergent solutions to DNA packaging challenges.

**TABLE 2:** Proteins featuring the double histone fold in non‐cellular life.

DHF variant or family	Encoded in	References	PDB IDs of experimentally‐determined structures (*virus family*)
Viral DHF proteins	*Heliotis zea* nudivirus 1 and *Nucleocytoviricota* species^a^	Greco, Fantucci, and de Gioia (2005), Liu et al. (2021), Valencia‐Sánchez et al. (2021)	7N8N, 7LV8, 7LV9 (*Marseilleviridae*)

^a^ Nucleocytoviricota species encoding for DHF proteins belong to the early‐branching superclades within the phylum (Irwin & Richards, 2024), such as marseilleviruses, iridoviruses and medusaviruses.

### 3.3 DHF proteins in bacteria

For many years, bacteria were thought to lack true histones. However, recent work has identified bacterial histone‐like proteins with clear structural similarity to archaeal and eukaryotic histones (Alva & Lupas, 2019; Hu et al., 2025). Among these, relatively rare examples of double histone folds have been identified, such as proteins from *Thermus thermophilus* * and *Aquifex aeolicus* (Qiu et al., 2006). Notably, these DHF proteins lack the DNA‐binding residues required for DNA binding and nucleosome‐like assembly (Schwab et al., 2025), therefore their functional role is unclear. BLAST searches evidence that homologs sharing high sequence identity with such bacterial DHF proteins (pairwise identities above 50%) are expressed in various bacteria kingdoms: in addition to Pseudomonadati—to which *A. aeolicus* belong—and Thermotogati, which include *T. thermophilus*, the other kingdoms that turned out to be relevant in such regard are Bacillati, as reported in Table 1. Finally, bacteria‐type histone doublets were found also in archaea (Qiu et al., 2006; Stevens & Warnecke, 2023); such occurrence is likely to depend on horizontal gene transfer, as hypothesized also in other cases of archaeal and bacterial histones (Schwab et al., 2024).

### 3.4 Eukaryotic single domain and multidomain proteins featuring the double histone fold

The discovery of a DHF in the N‐terminal domain of human Sos1 (hSos1, a guanine nucleotide exchange factor that acts on Ras GTPases) was unexpected (Sondermann et al., 2003), as such a protein is a cytosolic signaling protein, not a chromatin‐associated factor. Structural and computational analyses published more than 20 years ago demonstrated that this domain adopts a histone pseudodimer fold, despite limited sequence similarity to histones (Greco, Sacco, et al., 2005; Sondermann et al., 2003). Sos proteins are widespread among higher eukaryotes, as indicated by protein sequence searches carried out at the time of discovery of this DHF variant (Greco, Sacco, et al., 2005). Now, BLAST searches not only confirm such a picture, but further evidence that a Sos homolog with a conserved N‐terminal domain is expressed also in Capsaspora, a genus of protists that contains only one single species, that is, *Capsaspora owczarzaki* (see Table 1); notably, such a unicellular eukaryotic organism holds a pivotal phylogenetic role in studies of how animal multicellularity originated, being among the nearest single‐celled relatives of animals (Sebé‐Pedrós et al., 2016; Suga et al., 2013). In the multidomain Sos proteins, the conserved DHF domain has the role of mediating intramolecular interactions of functional relevance (Huang et al., 2019; Iversen et al., 2014; Sacco et al., 2012).

Bioinformatic studies (Greco, Sacco, et al., 2005) identified additional eukaryotic proteins containing DHFs, notably the Cca3 protein family (Hayashi et al., 1997)—now more commonly known as ankyrin repeat and BTB/POZ domain‐containing 2 (ABTB2) proteins—and related “similar to Cca3” (Cca3S) proteins; the latter share high sequence identity with the DHF domain sequences of the former (Greco, Sacco, et al., 2005). As implied by their denomination, ABTB2 proteins combine a DHF domain with ankyrin repeats and BTB/POZ domains, suggesting roles in protein–protein interactions and regulatory processes rather than constitutive chromatin organization. Actually, functional analyses of Cca3 proteins indicate preferential DNA binding in specific cell cycle states, implying a regulatory rather than structural role in chromatin biology (Greco, Sacco, et al., 2005; Hayashi et al., 1997; Miyake et al., 2026) (see also Section 4). Importantly, there is no evidence that these eukaryotic DHF proteins replace canonical histones in nucleosomes. As far as taxonomic distribution is concerned, previous literature (Greco, Sacco, et al., 2005) and BLAST searches indicate that they are common across the animal phyla, from sponges up to—as above implied—chordates.

As for Cca3S proteins, these are single‐domain proteins of unknown function; similarly to the case of the single‐domain SPT proteins (see the “Introduction” section and Table 1), they share a low/very low degree of sequence identity with nucleosome histones (ca. 25% and 30% identity to histones H2B and histone H2A, respectively, for the two contiguous histone fold sequences (Greco, Sacco, et al., 2005)).

As for the last variant of DHF proteins listed in Table 1, a recent targeted sequence and structure search revealed a surprisingly large extent of convergent evolution toward the DHF across diverse eukaryotic phyla, that is, Chordata, Arthropoda, Nematoda, Mollusca (Miyake et al., 2026). These results correspond to the identification of a large and diverse family of single‐domain DHF proteins that share a relatively high degree of sequence identity—60% or more—with nucleosome histones; as a whole, they constitute the abovementioned “High Sequence Identity with Histones,” DHF protein family. Such picture points at a possible role of HSIH‐DHF in eukaryotic chromatin organization.

## 4 FOCUS ON FUNCTIONAL ROLES OF THE DOUBLE HISTONE FOLD

A naturally recurring theme among DHF‐containing proteins is their potential to bind DNA. Structural models and analyses of electrostatic properties show that many DHFs expose positively charged surfaces analogous to those used by canonical histones to interact with the DNA phosphate backbone (Fahrner et al., 2001; Greco, Fantucci, & de Gioia, 2005; Greco, Sacco, et al., 2005; Liu et al., 2021).

In some cases, such as viral histone doublets, this DNA‐binding capability is likely essential for genome packaging (Liu et al., 2021); here, the use of a single‐chain pseudodimer may offer advantages in terms of stability and assembly efficiency.

In other contexts, including eukaryotic multidomain proteins such as ABTB2 proteins, DNA binding by the DHF assembly may be more transient or regulatory, contributing to processes such as transcriptional control, chromatin accessibility, or cell cycle–dependent chromatin states. Very recent studies on ABTB2 proteins indicate potential functions in protein complex assembly and transcriptional repression, with relevance across multiple cancer contexts (Gong et al., 2020; Iqbal et al., 2022).

In archaea and bacteria, DHFs may contribute to chromatin‐like structures that are simpler than eukaryotic nucleosomes but still capable of compacting DNA and regulating gene expression. It has to be underlined that in archaea, in particular, no examples of obligate heterodimerisation of histone proteins are known, to the best of our knowledge; as the presence of histone doublets leads to a pseudo‐heterodimerization, it is tempting to hypothesize that the occurrence of histone doublets might be an evolutive step toward the obligate heterodimerization of histones observed in eukaryotes. In this regard, the abovementioned fact that the very sparse recurrence of DHF among distantly related archaeal lineages includes also the Asgard archaea is particularly intriguing.

Interestingly, the sole example of DHF‐containing eukaryotic protein for which cytosolic localization is strictly confirmed, that is, the Sos protein family, is associated to the occurrence of histone folds that do not retain the electrostatic features essential for nucleic acids binding (Greco, Sacco, et al., 2005). In fact, in Sos the DHF domain has non‐canonical roles related to autoinhibition and protein–protein interactions, as also reported above in Section 3.

These considerations illustrate how the DHF can be repurposed as a versatile interaction module beyond the classical role of histones in chromatin.

## 5 DESIGN IMPLICATIONS: THE DHF AS A NATURAL BLUEPRINT FOR PROTEIN ENGINEERING

From a protein engineering perspective, the DHF exemplifies how evolution repeatedly converges on compact and robust architectural solutions to recurring molecular problems. The DHF converts an obligate intermolecular dimerization interface—characteristic of canonical histone heterodimers—into an intramolecularly encoded pseudodimer, thereby simplifying folding, assembly, and functional deployment, as noted also very recently (Irwin & Richards, 2024). Such a strategy has broad implications for protein design, particularly in the stabilization of interaction interfaces and the construction of modular DNA‐binding proteins.

Moreover, electrostatic surface analyses reveal that many DHFs expose positively charged regions analogous to those mediating DNA binding in canonical histones (Fahrner et al., 2001; Greco, Fantucci, & de Gioia, 2005; Greco, Sacco, et al., 2005; Liu et al., 2021). Importantly, this DNA‐binding capability appears to be an emergent property of the fold, arising from its geometry and residue distribution rather than from finely tuned sequence features.

This observation is consistent with broader principles of protein–DNA recognition, where fold geometry and electrostatics often play dominant roles over sequence‐specific interactions (Luscombe et al., 2000). From a design standpoint, the DHF could thus serve as a generic DNA‐binding scaffold, suitable for applications requiring non‐sequence‐specific DNA association, such as DNA condensation, chromatin‐inspired processes, or synthetic genome organization systems.

Regarding protein engineering in relation to synthetic chromatin biology, a particularly interesting perspective is offered by recent studies on viral DHF proteins (Irwin & Richards, 2024). In fact, the existence of linkers conjoining histone folds in viral histones was shown to facilitate nucleosome formation: viral H2A–H2B‐like and H3–H4‐like DHF couples, when joined together within the same protein sequence—thus giving place to a quadruplet—are able to efficiently promote the assembly of nucleosomes in E. coli. Notice that nucleosome assembly in eukaryotes typically requires chaperones (Burgess & Zhang, 2013); however, viral histone quadruplets formed nucleosomes in *Escherichia coli* without coexpression of eukaryotic proteins.

As far as in vitro nucleosome assembly is concerned, viral histone quadruplets promoted such process without salt‐gradient dialysis. As salt‐gradient dialysis is required for in vitro nucleosome reconstitution with eukaryotic histones, results obtained with viral histones imply that the latter could have a greater propensity for assembly relative to the former. However, very notably, cross‐linking of eukaryotic histones—giving place to artificial DHF proteins – was shown to lead to nucleosome formation within *E. coli* in a much easier way as compared to coexpression of separate, wild‐type nucleosomal histones in the same bacterial host (Irwin & Richards, 2024). This futher illustrates the powerful perspectives offered by DHF studies for the development of synthetic genome organization systems (see Figure 4).

Finally, the potential of DHF‐related investigation in the context of protein design may lead to fruitful interplays with preliminary evidences on chromatin reshaping by engineered histones (Jena et al., 2025). To date, the latter are based on point mutations in the context of investigations based on the development of large lentiviral libraries, whose long‐term target is the rational design of synthetic histones that can be used to engineer specified cell states. Notably, natural histone pseudodimers (see Figure 4) as well as artificial cross‐linking of separate histone folds may offer additional bases for advancements as well as novel degrees of freedom to tackle such a highly challenging scientific endeavor.

## 6 PERSPECTIVES AND OPEN QUESTIONS

Despite significant progress on the structure as well as on the biological role of DHF proteins (see Figure 4), many questions remain open. A key question regards whether (and in which additional cases, as compared to the known ones) DHF proteins form higher‐order assemblies analogous to nucleosome core. One may also wonder how widespread DHFs are in unexplored taxa, particularly in microbial dark matter. What regulatory mechanisms control their interaction with DNA in eukaryotic cells, especially in the case of DHF showing high sequence identity with nucleosome histones (HSIH‐DHF proteins)?

As implied in recent literature (Miyake et al., 2026), advances in structural prediction, particularly AlphaFold‐based approaches (Jumper et al., 2021), combined with functional genomics and biophysical studies, are likely to reveal many additional DHF‐containing proteins and clarify their roles. Beyond basic biology, the DHF may inspire the design of synthetic DNA‐binding proteins and novel tools for chromatin reshaping (Jena et al., 2025).

## 7 CONCLUSIONS

The DHF exemplifies the remarkable versatility of the histone fold as a structural motif. Once thought to be a rare anomaly, the DHF is now recognized as a recurrent solution for DNA binding and genome organization across all domains of life. Its study not only deepens our understanding of histone evolution but also highlights the broader principle that protein folds can be repeatedly reinvented to meet similar functional demands.

## AUTHOR CONTRIBUTIONS

*Conceptualization*: C.G., A.R., and T.M. *Writing—original draft preparation*: C.G., A.R., T.M., and U.C. *Supervision*: C.G. All authors have read and agreed to the published version of the manuscript.

## FUNDING INFORMATION

This research received no external funding.

## CONFLICT OF INTEREST STATEMENT

The authors declare no conflicts of interest.

## Data Availability

Data sharing not applicable to this article as no datasets were generated or analysed during the current study.

## Footnotes

* For the crystal structure of *T. thermophilus* DHF (PDB ID: 1WWI), no scientific article was published contextually with the deposition of coordinates in the Protein Data Bank, to the best of our knowledge.
